# Modulating Autophagy in Osteoarthritis: Exploring Emerging Therapeutic Drug Targets

**DOI:** 10.3390/ijms252413695

**Published:** 2024-12-21

**Authors:** Corina Andrei, Dragos Paul Mihai, George Mihai Nitulescu, Georgiana Nitulescu, Anca Zanfirescu

**Affiliations:** Faculty of Pharmacy, “Carol Davila” University of Medicine and Pharmacy, Traian Vuia 6, 020956 Bucharest, Romania; corina.andrei@umfcd.ro (C.A.); george.nitulescu@umfcd.ro (G.M.N.); georgiana.nitulescu@umfcd.ro (G.N.); anca.zanfirescu@umfcd.ro (A.Z.)

**Keywords:** osteoarthritis, autophagy, inflammation, disease-modifying treatments, cartilage, extracellular matrix

## Abstract

Osteoarthritis (OA) is a degenerative joint disease characterized by the breakdown of cartilage and the subsequent inflammation of joint tissues, leading to pain and reduced mobility. Despite advancements in symptomatic treatments, disease-modifying therapies for OA remain limited. This narrative review examines the dual role of autophagy in OA, emphasizing its protective functions during the early stages and its potential to contribute to cartilage degeneration in later stages. By delving into the molecular pathways that regulate autophagy, this review highlights its intricate interplay with oxidative stress and inflammation, key drivers of OA progression. Emerging therapeutic strategies aimed at modulating autophagy are explored, including pharmacological agents such as AMP kinase activators, and microRNA-based therapies. Preclinical studies reveal encouraging results, demonstrating that enhancing autophagy can reduce inflammation and decelerate cartilage degradation. However, the therapeutic benefits of autophagy modulation depend on precise, stage-specific approaches. Excessive or dysregulated autophagy in advanced OA may lead to chondrocyte apoptosis, exacerbating joint damage. This review underscores the promise of autophagy-based interventions in bridging the gap between experimental research and clinical application. By advancing our understanding of autophagy’s role in OA, these findings pave the way for innovative and effective therapies. Nonetheless, further research is essential to optimize these strategies, address potential off-target effects, and develop safe, targeted treatments that improve outcomes for OA patients.

## 1. Introduction

Osteoarthritis (OA) is the most common degenerative joint disease, affecting millions of people worldwide. It is primarily associated with aging, obesity, and joint injuries, and commonly affects the knees, hips, hands, and spine. As the cartilage in joints wears down, patients experience pain, stiffness, and swelling, which significantly reduce their mobility and overall quality of life [1]. The Global Burden of Disease project revealed that in 2019, 528 million people worldwide were affected by OA, with a 113% increase compared to 1990. The knee was the most commonly impacted joint, followed by the hip and hand, and 344 million people experienced moderate to severe symptoms. With aging populations, obesity, and injuries on the rise, OA prevalence is expected to keep growing globally [1].

Globally, OA remains a major cause of disability and is responsible for substantial economic burdens due to healthcare costs and lost productivity. The increasing prevalence of OA, particularly in older adults and those with obesity, highlights the urgent need for effective prevention and treatment strategies [2]. Current treatments for OA primarily focus on symptom management, especially pain relief, and lack effective disease-modifying therapies. Standard treatments, such as nonsteroidal anti-inflammatory drugs, acetaminophen, and opioids, provide only temporary pain relief and do not address the underlying joint degeneration. These drugs also carry risks of adverse effects, including gastrointestinal and cardiovascular complications with NSAIDs, and addiction potential with opioids [3,4]. While intra-articular injections of corticosteroids or hyaluronic acid offer short-term relief, they do not modify the disease’s progression, and emerging therapies, such as biologics targeting inflammatory pathways, are still in early research stages, with clinical trials revealing a mediocre effectiveness [5]. Regenerative approaches, including stem cell therapies and growth factors, show promise but require further clinical validation [6]. Thus, there remains a significant unmet need for therapies that not only alleviate symptoms but also slow or reverse the progression of OA.

Autophagy is a crucial cellular process responsible for degrading and recycling damaged organelles and proteins, maintaining cellular homeostasis. This process involves the formation of autophagosomes, which encapsulate cellular debris and transport it to lysosomes for degradation. Autophagy plays a vital role in responding to cellular stress, such as nutrient deprivation or damage, by clearing malfunctioning components and preventing their toxic accumulation. This function is essential for protecting cells from stress and maintaining homeostasis [7]. Dysregulation of autophagy has been linked to various diseases, including cancer, neurodegenerative disorders, and cardiovascular diseases. For instance, autophagy’s role in removing damaged mitochondria (mitophagy) helps prevent oxidative stress and cellular dysfunction. Additionally, the recycling aspect of autophagy supports cellular energy demands during metabolic stress, ensuring survival in adverse conditions [8].

In OA, the decline of autophagy is a key factor contributing to disease progression, leading to chondrocyte dysfunction and cartilage degradation [9,10,11,12]. This reduction in autophagy results in the accumulation of damaged cellular components, impairing chondrocyte survival and accelerating cartilage breakdown [13]. Autophagy plays a crucial role in maintaining the balance between chondrocyte survival and inflammation by regulating apoptosis and reactive oxygen species (ROS) production [14]. When autophagy is disrupted, it exacerbates the production of inflammatory cytokines and cartilage-degrading enzymes, further deteriorating the cartilage matrix [15]. The role of autophagy-related genes in OA and their association with immune infiltration in articular cartilage were previously explored in clinical specimens from OA patients and by applying bioinformatics tools on freely available microarray datasets. The performed analysis identified 24 downregulated and 5 upregulated autophagy-related genes, with hub genes including *CDKN1A*, *DDIT3*, *FOS*, *RELA*, *MAP1LC3B*, *HSPA5*, *HSPA8*, and *MYC*, which were validated as significantly downregulated in OA and linked to pathways involving autophagy, mitophagy, and inflammation. Additionally, a negative correlation between DDIT3 expression and immature dendritic cells, and a positive correlation between FOS expression and eosinophils were also revealed, suggesting autophagy-related genes influence immune cell infiltration in OA [16].

Enhancing autophagy could represent a therapeutic strategy for preventing OA progression by improving chondrocyte survival and maintaining cartilage integrity. The purpose of this review is to explore the therapeutic potential of targeting autophagy in OA. While numerous studies have identified the role of autophagy in cellular homeostasis and its dysfunction in OA progression, few have comprehensively examined the emerging therapeutic strategies aimed at modulating autophagic pathways for disease modification. This review seeks to fill this gap by summarizing recent advancements in pharmacological approaches that enhance autophagy. It also emphasizes the need for further clinical translation of these findings, as many studies remain in the preclinical phase, leaving a critical gap between laboratory discoveries and practical OA therapies.

## 2. Materials and Methods

This narrative review focused on possible pharmacological targets regulating autophagy as a treatment for OA. The comprehensive analysis of literature was conducted by searching major scientific databases, including PubMed (accessed on 1–2 October 2024), Scopus (accessed on 4–5 October 2024) and Web of Science (accessed on 7–8 October 2024), for relevant peer-reviewed articles published in English up until October 2024. Search terms included combinations of the following keywords: “osteoarthritis” AND “autophagy” OR “mitophagy” AND “chondrocyte apoptosis” OR “cartilage degradation” OR “extracellular matrix degradation” OR “inflammation” OR “oxidative stress” OR “bone remodeling”.

The inclusion criteria were studies that:

- Investigated the role of autophagy in OA,
- Explored molecular pathways of autophagy, and
- Discussed therapeutic interventions aimed at modulating autophagy in OA.

Exclusion criteria were:

- Studies not focused on autophagy or OA.
- Publications not in English.
- Conference abstracts or review articles lacking original research.

Gray literature (e.g., preprints and theses) and references from selected articles were screened manually for any additional relevant studies. The narrative synthesis of the results focused on summarizing key molecular mechanisms linking autophagy and OA progression and evaluating emerging pharmacological interventions targeting autophagic pathways.

No human or animal intervention studies were conducted as part of this review, and therefore, no ethical approvals were required.

## 3. Role of Autophagy in Various Stages of OA

### 3.1. Early Phase

Early-stage OA is often accompanied by low-grade synovitis, characterized by elevated levels of pro-inflammatory cytokines such as interleukin (IL)-15, tumor necrosis factor (TNF)-α, and IL-6 [17]. Notably, increased concentrations of both pro-inflammatory (IL-7, IL-12, IFN-γ) and anti-inflammatory (IL-10, IL-13) cytokines, along with growth factors SCGF-β (stem cell growth factor β) and VEGF (vascular endothelial growth factor) in synovial fluid samples were strongly correlated with knee pain intensity, while significant correlations were established between OA severity and levels of IL-6, IL-8, IFN-γ (interferon gamma), SCGF-β, VEGF, and CXCL1 (chemokine (C-X-C motif) ligand 1) [18]. During early OA, chondrocytes exhibit increased proliferation, which represents a compensatory mechanism to repair the damaged cartilage matrix. Subsequently, they increase their synthetic activity to repair damaged tissue, leading to a compensatory increase in collagen types I and II levels. However, collagen fibrils become disorganized, and proteoglycan levels are decreased, both elements being critical for maintaining cartilage structure and function [19].

Inflammation increases ROS levels. The oxidative stress damages mitochondria, causing them to malfunction. In response, mitophagy is activated, aiming to remove dysfunctional mitochondria and protect the cell [20]. In response to stress, Unc-51-like autophagy activating kinase 1 (ULK1), beclin1, and microtubule-associated protein 1A/1B-light chain 3 (LC3) are activated, initiating the formation of autophagosomes [21]. At this point, autophagy plays a critical role in maintaining chondrocyte homeostasis and responding to initial stressors such as mechanical stress and early inflammatory signals. Autophagy prevents the accumulation of damaged organelles and proteins from minor cartilage damage, thus preserving chondrocyte function and viability. Interestingly, a dual role of autophagy was investigated by Chang et al. in chondrocytes. According to their findings, autophagy induced with mTOR inhibitor rapamycin showed an in vitro cytoprotective effect by preventing the accumulation of subdiploid cells in young chondrocytes. In contrast, rapamycin-induced autophagy resulted in cell death in OA chondrocytes, while autophagy inhibition promoted chondrocyte survival. Moreover, the same team showed that, unexpectedly, the expression levels of autophagy-related proteins were increased in tissue samples from OA patients [22]. In the early phase, autophagy serves as a critical adaptive response in OA, capable of promoting cell survival and preventing early apoptosis under stress [23].

### 3.2. Progressive Phase

At this point, several key molecular and cellular changes occur, reflecting ongoing cartilage degradation and joint destruction. An imbalance between anabolic and catabolic processes occurs. Further cartilage degradation is associated with a notable rise in a di-sintegrin and metalloproteinase with thrombospondin motifs (ADAMTS) and matrix metalloproteinases (MMPs), such as MMP13 [24]. Pro-inflammatory cytokines, such as IL-1, TNF-α, and IL-6, further stimulate the production of MMPs and other enzymes that contribute to cartilage breakdown. The involvement of innate immune system components, including pattern recognition receptors and complement factors, further drives this inflammatory cascade [24,25].

Subchondral bone undergoes sclerosis and the formation of osteophytes, while synovitis persists [26]. The upregulation of RUNX2 (runt-related transcription factor 2), a transcription factor that promotes osteoblast differentiation and bone formation, was associated with osteophyte formation. RUNX2 can be regarded as a critical driver of OA progression due to its role in mediating chondrocyte hypertrophy, extracellular matrix breakdown, and inflammation and stress response [27]. Interestingly, homozygous knockout of RUNX2 accelerated OA progression in mice, highlighting the critical role of RUNX2 in maintaining the balance between anabolic and catabolic activities in cartilage, since complete loss of transcriptional activity leads to impaired cartilage repair mechanisms [28]. Additionally, mitochondrial dysfunction and oxidative stress are further enhanced during the progressive phase [29].

Chondrocytes undergo apoptosis due to mechanical stress, inflammatory signals, and oxidative stress, leading to impaired cartilage repair capacity [30]. Initially, autophagy functions as a protective mechanism in response to cellular stress, aiding in mitigating the accumulation of damaged organelles and misfolded proteins. Previous studies demonstrated that autophagy-related markers (e.g., LC3 and beclin1) are upregulated in the early stages of OA, correlating with reduced chondrocyte apoptosis and cartilage damage. However, as OA progresses, the effectiveness of autophagy diminishes due to sustained cellular stress and inflammatory insults, as evidenced by decreased autophagy marker expression in advanced OA cartilage. Excessive or dysregulated autophagy may also contribute to cellular dysfunction, setting the stage for further cartilage degradation. For instance, dysregulated autophagy promotes aberrant protein degradation and cellular energy deletion, exacerbating chondrocyte death and cartilage erosion. While autophagy is essential for cellular cleanup and turnover, its dysregulation in OA may promote autophagic cell death rather than survival, contributing to the transition from cartilage damage to overt cartilage erosion and subchondral bone remodeling. Therefore, these findings illustrate the dual role of autophagy in OA: while crucial for cartilage homeostasis, its dysregulation can mark the transition from cartilage damage to irreversible joint degradation [31,32].

### 3.3. End-Stage Phase of OA

At the end-stage phase, the pathology worsens due to severe cartilage loss, matrix calcification, and heightened apoptosis, marking an advanced state of joint destruction and loss of function [33,34]. As the disease progresses to more advanced stages; however, the role of autophagy shifts. Instead of effectively removing damaged mitochondria, excessive autophagy occurs, resulting in the accumulation of autophagosomes without proper degradation. This overwhelms cellular systems, leading to a blockage of autophagic flux [35]. Dysfunctional autophagy signals also activate apoptosis. Autophagic stress causes the release of factors like apoptosis-inducing factor (AIF) and the serine protease Omi/high temperature requirement protein A2 (HtRA2) from damaged mitochondria [30]. This leads to caspase-independent apoptosis, where autophagy and apoptosis overlap, and chondrocyte death is accelerated [36]. As OA progresses, chondrocytes undergo both apoptotic and autophagic cell death. Autophagy initially serves as a protective mechanism, but in severe OA, excessive or dysfunctional autophagy leads to cell death. In OA chondrocytes, a combination of apoptosis and autophagy has been observed, both cell processes operating simultaneously [37]. Thus, autophagy may transition from a survival mechanism to a mechanism facilitating programmed cell death, reflecting a response to irreversible cellular damage and severe oxidative stress. This phenomenon is supported by a study published by Yan et al., who indicated that autophagic processes drive the production of LC3-positive calcified extracellular vesicles (CEVs), which serve as nucleation sites for cartilage calcification. CEVs are involved in the pathological calcification observed in OA, marking a shift in the role of autophagy from protective to maladaptive as it contributes to the progression of joint damage [38].

These distinct roles of autophagy in the different phases of OA underline its dual nature depending on the context and stage of the disease. While enhancing autophagy may be beneficial in younger cells or early stages of cartilage deterioration by mitigating cellular stress and preventing apoptosis, its role in mid-to-late-stage OA is more complex. Most OA patients recognize their disease at advanced stages typically accompanied by pain and significant damage, when autophagy might already be dysregulated. At this point, elevating autophagy indiscriminately could exacerbate autophagic cell death, while its complete suppression may hinder necessary repair mechanisms. Hence, stage-specific and context-dependent modulation of autophagy is highly important, with careful modulation being required in OA-affected chondrocytes to prevent exacerbation of the disease through autophagic cell death. Emerging evidence suggests that targeting specific forms of autophagy (e.g., mitophagy) can selectively address cellular dysfunction without activating maladaptive pathways [39,40]. Additionally, small molecules, such as mTOR inhibitors and AMPK activators, were investigated for their ability to restore autophagic balance and mitigate cartilage degradation in OA (see Section 5) [41,42,43]. A further understanding of these mechanisms is crucial for developing targeted therapies that can effectively modulate autophagy to treat or slow the progression of OA, particularly at the mid-to-late stages of the disease [31].

## 4. Key Molecular Pathways of Autophagy in OA

### 4.1. Mechanistic Target of Rapamycin (mTOR) Pathway

#### 4.1.1. mTOR in OA

mTOR is a serine/threonine kinase that acts as a central regulator of cellular homeostasis, regulating cell growth, proliferation, and survival in response to environmental cues such as growth factors, nutrients, and energy status. This protein can be part of two functionally distinct complexes due to its interaction with distinct sets of proteins, which modify its function depending on the complex [44].

Thus, mTORC1 plays a crucial role in promoting anabolic metabolism while inhibiting catabolic pathways, including autophagy. As a key negative regulator of autophagy, mTORC1 inhibits its initiation by phosphorylating essential components of the autophagic machinery, such as ULK1 and autophagy-related protein (ATG)13. When nutrients are abundant, mTORC1 activity is elevated, inhibiting autophagy. Conversely, under conditions of nutrient deprivation or cellular stress, the inhibition of mTORC1 induces autophagy, allowing cells to recycle damaged organelles and proteins and facilitating cellular survival [42].

mTORC2 plays a crucial role in regulating cell survival and cytoskeletal organization, making it an important component in cellular stress response. Unlike mTORC1, which primarily controls autophagy, mTORC2 is involved in maintaining the structural integrity of cells by modulating actin cytoskeleton dynamics. It influences cell migration, adhesion, and overall stability. By regulating proteins like Rho GTPases and protein kinase B (Akt), mTORC2 helps cells adapt to environmental stress, promoting resilience and survival [45].

The interplay between mTOR and autophagy is particularly significant in the pathogenesis of OA, since mTOR is involved in maintaining chondrocyte health and cartilage integrity. In OA cartilage, the reduced autophagic activity associated with excessive mTORC1 signaling results in chondrocyte apoptosis and the disruption of extracellular matrix homeostasis. This reduction in autophagy contributes to the accumulation of damaged cellular waste and promotes chondrocyte apoptosis, exacerbating cartilage degeneration and contributing to the inflammatory environment of OA joints. Cartilage-specific ablation of mTOR results in increased autophagy signaling and significant protection in animal models of OA associated with a significant reduction in articular cartilage degradation, cell death, and synovial fibrosis [46].

PP2Ac, the catalytic subunit of protein phosphatase 2A, plays a crucial role in regulating autophagy during osteoclastogenesis in OA through its interaction with the mTOR pathway. mTORC1 inhibition was found to increase PP2Ac expression, suggesting a feedback mechanism between mTOR activity and PP2Ac levels. PP2Ac, in turn, regulates autophagy by enhancing the dephosphorylation of ULK1, specifically at the Ser637 residue, which is critical for autophagy initiation. This relationship indicates that PP2Ac may mediate autophagy in part as a downstream effector of mTOR [47].

#### 4.1.2. Upstream Regulators of the mTOR Pathway

Several signaling pathways and molecules contribute to OA progression by regulating mTOR-induced autophagy.

Regulated in development and DNA damage-response 1 (REDD1), an endogenous mTOR inhibitor, is crucial for cartilage health. In healthy human and mouse cartilage, REDD1 levels are high but significantly decrease in aged and OA tissues. In chondrocytes, REDD1 knockdown increases mTOR activity, while its overexpression suppresses it, confirming its role as a negative regulator of this pathway. Reduced REDD1 expression with aging leads to mTOR overactivation and defective autophagy, contributing to OA progression [48].

The phosphoinositide 3-kinase (PI3K)/AKT pathway enhances mTOR activity. Growth factors binding to specific receptors activate PI3K, converting phosphatidylinositol-4,5-bisphosphate into phosphatidylinositol-3,4,5-trisphosphate [49]. PIP3 recruits AKT, which inhibits Tuberous Sclerosis Complex (TSC1/TSC2), a key negative regulator of mTORC1 [50,51]. Reduced PI3K/AKT activity in OA-affected cartilage exacerbates cellular damage and inflammation. Amino acids such as arginine and leucine also activate Rag GTPases, further stimulating mTORC1 and inhibiting autophagy [52,53].

On the other hand, AMP-activated protein kinase (AMPK), an energy sensor, inhibits mTOR during low energy states, promoting autophagy to maintain cellular homeostasis. When cellular energy levels are low (high AMP ratio), AMPK inhibits mTORC1 by directly phosphorylating and activating TSC2 or by phosphorylating Raptor, a component of mTORC1, leading to mTORC1 inhibition. This promotes autophagy as a compensatory response to maintain energy balance [41].

While in normal articular chondrocytes, AMPKα is robustly phosphorylated, in articular cartilage from OA-affected human knees and joint tissue from OA mouse models, AMPKα phosphorylation at T172 is significantly reduced [54]. Additionally, AMPK activity was notably inhibited in chondrocytes exposed to inflammatory cytokines and mechanical injury. siRNA-induced AMPK depletion in chondrocytes worsened the catabolic response to IL-1β and TNF-α [55]. These findings suggest that AMPK activity is essential for maintaining cartilage homeostasis and preserving the chondrocyte phenotype.

Hypoxia negatively regulates mTORC1 through REDD1, which promotes the activation of TSC1/TSC2, leading to mTORC1 inhibition. This allows autophagy to be activated during low oxygen conditions [56]. Hypoxia, or low oxygen levels, plays a critical role in regulating autophagy in OA. Articular cartilage is an avascular tissue, meaning that chondrocytes often exist in a naturally low-oxygen environment. Under normal conditions, hypoxia induces autophagy as a survival mechanism, helping chondrocytes cope with cellular stress by recycling damaged organelles and maintaining homeostasis. However, in OA, this adaptive response is disrupted. Hypoxia-inducible factor 1-alpha (HIF-1α), a key regulator of autophagy under hypoxic conditions, becomes dysregulated, impairing the autophagic process [57]. Elevated levels of HIF-1α were found in both human and animal models of OA, particularly in the synovial fluid and cartilage, and were correlated with disease severity. The upregulation of HIF-1α in OA chondrocytes, especially under catabolic stress and inflammatory conditions, suggests that it helps chondrocytes adapt to the hypoxic environment by promoting energy metabolism and matrix synthesis [58]. However, while this adaptive response initially aids cell survival, chronic overactivation of HIF-1α can exacerbate cartilage degradation by stimulating the production of matrix-degrading enzymes. This overactivity can disrupt the balance between matrix synthesis and degradation, leading to increased breakdown of cartilage over time [59]. Thus, while HIF-1α plays a protective role in maintaining chondrocyte viability under stress, its prolonged activation also drives the degenerative processes characteristic of OA. Another regulator factor of autophagy is HIF-2α. Compared to HIF-1α, HIF-2α expression is increased in healthy cartilage and decreased in OA cartilage. In a study, an inverse correlation between HIF-2α and HIF-1α expression in chondrocytes was observed. Reduced levels of HIF-2α are correlated with increased expression of HIF-1α and excessive stimulation of autophagy. Therefore, HIF-2α may protect cells from inappropriate autophagy induced by HIF-1α [57].

The p53 pathway plays a complex role in the regulation of autophagy via mTOR. As a key tumor suppressor, p53 regulates cell cycle and apoptosis, but it also has dual effects on autophagy depending on its cellular localization. In the nucleus, p53 promotes autophagy, aiding in the removal of damaged organelles and maintaining cellular homeostasis. However, in the cytoplasm, p53 can inhibit autophagy, leading to increased cellular stress [60]. In OA, oxidative stress and inflammation can activate the p53 pathway, contributing to chondrocyte apoptosis and reduced autophagic activity, which worsens cartilage degradation. Dysregulated autophagy, influenced by p53, may impair the chondrocytes’ ability to clear damaged proteins and organelles, leading to further cell death and extracellular matrix breakdown, thus accelerating OA progression [61]. This suggests that the p53-autophagy interaction plays a significant role in OA pathogenesis, balancing between cell survival and death in response to joint stress and inflammation.

The studies collectively show that p53 levels are elevated in OA tissues and are associated with disease severity. Overexpression of p53 is observed in both synovial tissue and cartilage, with higher levels found in more advanced stages of OA. This increase in p53 expression appears to contribute to chondrocyte apoptosis and the disruption of normal cartilage homeostasis. Additionally, p53 is negatively correlated with autophagy, suggesting that its upregulation may impair the protective autophagic response in chondrocytes, leading to further cartilage degradation and progression of OA [62].

### 4.2. Beclin1 and LC3 Pathways

#### 4.2.1. Beclin1 and LC3 in OA

These proteins are central to the autophagy machinery, being directly involved in the formation and maturation of autophagosomes, with beclin1 being a key initiator of the former, and LC3 being involved in the latter. LC3 is conjugated to phosphatidylethanolamine to form LC3-II, which is essential for the elongation and closure of autophagosomes. The LC3 pathway operates downstream of beclin1 [63].

By promoting autophagy, beclin1/LC3 ensures the removal of damaged cellular components. In OA, disruptions in this process can contribute to the accumulation of cellular waste in chondrocytes, accelerating cartilage degeneration [64].

In vivo studies involving rabbit models of OA have demonstrated that intra-articular injection of collagenase leads to decreased beclin1 levels and subsequent cartilage degeneration [65]. In the superficial zone of cartilage affected by mild OA, but not in the middle and deep zones, the protein levels of expression of beclin1 and LC3 are reduced. In mouse OA knee joints, the decrease in beclin1 and LC3 was associated with glycosaminoglycan loss, and increased PARP p85 expression [66].

In cartilage tissue from patients with OA, beclin1 expression levels are significantly decreased, while its overexpression inhibits chondrocyte apoptosis and downregulates extracellular matrix metabolism in OA [64]. YAP1, a transcriptional coactivator, interacts with beclin1 and inhibits its function, leading to decreased autophagy and promoting OA progression [67].

#### 4.2.2. Upstream Regulators of the Beclin1/LC3 Pathway

mTOR modulates the activity of beclin1 by controlling the initiation of autophagy through upstream signaling [64,68]. However, beclin1 plays a significant role in OA through pathways that are independent of mTOR [69].

Beclin1 forms part of the class III PI3K complex, which is essential for the nucleation of the autophagosome [70]. This pathway operates independently of mTOR and is vital for cellular maintenance [71].

Another regulating pathway involves the interaction of beclin1with Bcl-2 and Bcl-xL, which are anti-apoptotic proteins. Normally, Bcl-2 and Bcl-xL bind to beclin1, preventing autophagy [72]. However, under stress conditions common in OA, this interaction is disrupted, allowing beclin1 to initiate autophagy and helping to preserve cartilage integrity. When this pathway is dysregulated, autophagy is impaired, leading to further cartilage damage in OA [73].

AMPK modulates beclin1 function, especially under conditions of energy stress. It activates autophagy by directly phosphorylating at Ser93, Ser90 [74,75], and Thr388 residue of beclin1 [76], which enhances its interaction with the Class III PI3K complex and promotes autophagosome formation. This phosphorylation process helps dissociate beclin1 from its inhibitors, like Bcl-2, thereby facilitating autophagy [77]. In OA, the diminished activity of AMPK leads to less beclin1 activation, further reducing autophagic processes. This deficiency in the AMPK-beclin1 pathway results in increased cellular stress and inflammation, contributing to the degeneration of cartilage typical in OA [15,78]. Conversely, activation of AMPK can enhance beclin1 levels and promote autophagy, thereby mitigating the catabolic responses triggered by pro-inflammatory cytokines such as IL-1β [79].

Oxidative stress, caused by the accumulation of ROS, triggers cellular damage and stress responses, partially by increasing the expression of beclin1. Several studies have shown that oxidative stress can enhance beclin1 expression, leading to increased autophagy, as cells attempt to cope with the damage caused by ROS [80,81,82].

Oxidative stress influences beclin1 through both transcriptional regulation and post-translational modifications. It can increase the transcription of autophagy-related genes, including beclin1, by activating stress-responsive pathways like NF-κB and p53 [83,84]. Additionally, it can modify beclin1 activity through post-translational changes, such as phosphorylation or interactions with regulatory proteins (e.g., Bcl-2) [85,86]. These modifications can either inhibit or enhance beclin1’s ability to promote autophagy, depending on the cellular environment and specific conditions [87].

While mTOR is a central regulator of autophagy, both beclin1 and LC3 are modulated by various other upstream factors such as Bcl-2, AMPK, p53, and oxidative stress. These pathways allow autophagy to be regulated by diverse signals, including energy status, growth factors, stress, and cell death signals, offering multiple layers of control beyond mTOR.

### 4.3. ROS

ROS are pivotal contributors to the pathogenesis of OA, arising from various cellular and environmental sources. The NADPH oxidase family of enzymes, which are expressed in chondrocytes and synovial cells, catalyzes the production of superoxide anions, leading to oxidative stress and damage of structural biomolecules within the joint [88]. Mitochondrial dysfunction also plays a significant role in ROS generation; under conditions of oxidative stress, mitochondria can become overwhelmed, resulting in increased ROS production and subsequent cellular damage. Mitochondrial ROS can signal the removal of damaged mitochondria through mitophagy, protecting cells from oxidative stress. However, persistent ROS generation overwhelms mitophagic capacity, contributing to chondrocyte apoptosis [89]. Other contributing factors include mechanical stress on the joints, which can exacerbate ROS generation through inflammatory pathways, further promoting chondrocyte senescence and apoptosis. The accumulation of ROS not only disrupts cartilage homeostasis but also activates pro-inflammatory signaling pathways, creating a vicious cycle that accelerates OA progression [90]. Moreover, the inflammatory environment within OA-affected joints significantly contributes to elevated ROS levels. Inflammatory cytokines such as IL-1β stimulate chondrocytes and macrophages to produce more ROS, intensifying oxidative stress and leading to further cartilage degradation. Synovitis is also associated with increased levels of ROS and contributes to the overall deterioration of joint health. The interplay between ROS production and inflammation highlights the complexity of OA pathogenesis, where excessive oxidative stress not only damages cartilage but also perpetuates inflammatory responses that drive disease progression [90,91].

As previously discussed, ROS can also directly influence autophagy through the regulation of transcription factors, such as p53 and NF-κB, which upregulate the expression of autophagy-related genes (beclin1 and LC3). Oxidative stress-mediated activation of p53 can enhance autophagy as a cellular survival mechanism, while NF-κB can either promote or inhibit autophagy depending on the cellular milieu. Furthermore, ROS can induce autophagy through post-translational modifications of proteins critical for autophagy initiation. For instance, the oxidation of cysteine residues in autophagy-regulating proteins ULK1 and AMPK can promote autophagosome formation. Furthermore, oxidation or phosphorylation of proteins in the BCL-2 family can disrupt the inhibitory interaction between Bcl-2 and beclin1, allowing unbound beclin1 to initiate autophagy [92].

ROS-induced autophagy plays a dual role in OA. In the early stages, autophagy induced by ROS acts as a protective mechanism, facilitating the removal of damaged organelles and proteins to maintain homeostasis, limiting oxidative stress, and delaying cartilage degradation. In advanced stages, chronic oxidative stress may dysregulate autophagy, leading to excessive autophagic activity and autophagic cell death. Therefore, cartilage degradation and synovial inflammation are exacerbated; hence, modulating ROS levels and their impact on autophagy could provide therapeutic benefits in OA. Antioxidant therapies could reduce ROS production and restore autophagic balance, preventing both chondrocyte apoptosis and cartilage degradation. For instance, targeting ROS generation by LDHA (lactate dehydrogenase A) has been proposed as a therapeutic approach to inhibit excessive autophagy and its deleterious effects on cartilage [90].

### 4.4. Noncoding RNAs (ncRNAs) Regulating Autophagy

Noncoding RNAs (ncRNAs) are RNA molecules that do not code for proteins but regulate gene expression. Based on their length, they are categorized into small noncoding RNAs (20–30 nucleotides), which include microRNAs (miRNAs), small interfering RNAs (siRNAs), and PIWI-interacting RNAs (piRNAs), and long noncoding RNAs (lncRNAs) (over 200 nucleotides). miRNAs regulate gene expression post-transcriptionally by binding to mRNA, leading to its degradation or translation inhibition. siRNAs are involved in gene silencing, often through RNA interference. Other types of functionally significant ncRNAs include circular RNAs (circRNAs), small nuclear RNAs (snRNAs), and small nucleolar RNAs (snoRNAs), which are recognized for their regulatory roles in various cellular processes. For instance, ciRS-7 (circular RNA sponge for miR-7) has been involved in modulating gene expression by acting as miRNA sponges and influencing pathways relevant to disease pathogenesis. LncRNAs, on the other hand, are involved in various regulatory processes, including chromatin modification, transcriptional regulation, and sponging of other RNA molecules to control gene expression [93].

Several miRNAs are linked to autophagy dysregulation in OA, contributing to both chondrocyte viability and cartilage degradation, depending on the involved molecular pathways. Inflammatory stimuli such as IL-1β and IL-17A result in the formation of miR-7, leading to autophagy suppression via the PI3K/AKT/mTOR pathway, disrupting chondrocyte homeostasis, and accelerating cartilage breakdown [94]. Similarly, miR-146a-5p is significantly upregulated in OA cartilage, while NUMB is downregulated and negatively regulated by miR-146a-5p. By modulating various apoptosis and autophagy-related proteins, miR-146a-5p promotes chondrocyte apoptosis and inhibits autophagy. Increasing NUMB levels can reverse these effects, preserving chondrocyte viability and cartilage integrity [95]. On the other hand, miR-335-5p is significantly downregulated in OA chondrocytes, impairing autophagy, while its overexpression restores autophagy by enhancing the expression of key autophagy-related factors, such as beclin1, ATG5, and ATG7, promoting cellular survival [96]. miR-155 inhibits autophagy by targeting multiple autophagy-related genes, including ULK1, ATG3, and LC3, leading to impaired autophagic flux in OA chondrocytes. This dysfunction in autophagy is closely associated with the progression of OA [97].

LncRNA Small Nucleolar RNA Host Gene 1 (SNHG1) enhances chondrocyte viability and reduces apoptosis through the activation of the PI3K/Akt signaling pathway and suppression of autophagy. Overexpression of SNHG1 increases the expression of Bcl-2 [98].

CircRHOT1 is upregulated in OA tissues, where it suppresses autophagy markers (LC3 and beclin1) while enhancing CCND1 expression by sponging miR-142-5p. This modulation impacts chondrocyte viability and ECM components, suggesting that targeting circRHOT1 could alter OA progression [99]. On the other hand, reduced circRNA ciRS-7 expression in OA could lead to increased miR-7 activity, promoting chondrocyte apoptosis and cartilage degradation. Since ciRS-7 modulates inflammatory cytokine release, its downregulation leads to enhanced inflammation in IL-1β-stimulated chondrocytes, exacerbating joint damage. Moreover, the downregulation of ciRS-7 in OA inhibits autophagy, contributing to the progression of OA by disrupting cellular homeostasis [93,94].

In conclusion, ncRNAs play crucial roles in regulating autophagy and other key cellular processes in OA. miRNAs such as miR-7 contribute to cartilage degradation by inhibiting autophagy, while others like miR-335-5p promote autophagy when overexpressed, providing protection. Additionally, lncRNAs like SNHG1 and circRHOT1 further modulate autophagy and chondrocyte survival, influencing OA progression. Understanding these ncRNA pathways provides valuable insights into potential therapeutic strategies aimed at restoring autophagy and slowing the progression of OA.

## 5. Emerging Therapeutic Drug Targets for Modulating Autophagy in OA

Various pharmacological interventions targeting mTOR-induced autophagy have demonstrated promising effects on OA progression, primarily by reducing cartilage degradation, inflammation, and enhancing autophagy. Several studies have focused on inhibiting the PI3K/AKT/mTOR pathway to restore autophagic processes. For instance, four-octyl itaconate [100], β-ecdysterone [101], and mulberroside A [102], when administered via intra-articular injections in animal models, have significantly reduced the expression of MMP3, MMP13, and ADAMTS5, while increasing levels of structural proteins like collagen II and aggrecan, which collectively led to reduced cartilage destruction and inflammation. Similar effects were observed for shikonin in IL-1β-induced OA chondrocytes [103] as well as for FBXO21-specific knock-down shRNA [104]. Alternatively, treatments, such as calcitriol [105], and metformin [43], modulate the AMPK/mTOR signaling pathway. Bilobalide [106] and glabridin [107] ameliorate pain and cartilage damage via the mTOR pathway. These interventions promote autophagy by enhancing the AMPK signaling and reducing mTOR activity, which results in reduced proteoglycan loss, cartilage damage, and inflammation.

AMPK plays a crucial role in OA by regulating autophagy in chondrocytes [108]. Under stress conditions, AMPK is activated and regulates ULK1 activity by phosphorylating Ser317 and Ser377 residues on ULK1. Activated ULK1 subsequently promotes autophagy [109]. Additionally, activation of the AMPK/ULK1 pathway reduced oxidative stress-induced apoptosis and ER stress in chondrocytes and enhanced autophagy independently of mTOR [110]. Therefore, improving autophagic flux and reducing apoptosis via the AMPK/ULK1 pathway may contribute to the maintenance of articular cartilage integrity. Trehalose is a promising therapeutic target that activates autophagy via the AMPK/ULK1 pathway [111].

Notably, miRNA-based treatments like miRNA-100-5p [112] and miR-146a-5p antagomir [95] have also demonstrated protective effects by modulating mTOR signaling, improving autophagy, and ameliorating cartilage degeneration. Targeting the TSC2/RHEB/mTOR signaling pathway with quercetin alleviated knee OA by modulating autophagy and reducing cartilage degradation [113]. Inhibition of the NF-κB and STAT3 signaling pathways using alantolactone [114] or celastrol [115] reduced inflammation and promoted autophagy, thereby reducing cartilage degradation and pain. Pharmacological interventions targeting various autophagy-related pathways are listed in Table 1.

Early in OA, autophagy is upregulated, as indicated by the elevated expression of beclin1 and the conversion of LC3B-I to LC3B-II, signaling autophagosome formation. However, as OA progresses, autophagy dysfunction leads to chondrocyte apoptosis and increased matrix degradation [65].

Metformin is an antihyperglycemic drug of the biguanide class, approved for the treatment of type 2 diabetes mellitus as monotherapy or in combination with other drugs [117]. As an antihyperglycemic, metformin acts by using several mechanisms, such as reducing intestinal glucose absorption [118], or inhibiting hepatic gluconeogenesis by activating AMPK [119,120]. In recent years, research has focused on evaluating the anti-aging [121], immunomodulatory [122], cholesterol-lowering [123], anti-inflammatory [124] and analgesic [125] effects of metformin. In OA, metformin mediates autophagy by activating AMPK, which inhibits the mTOR signaling pathway [43] and increases SIRT1 expression [116]. Moreover, metformin decreased MMP expression, proteoglycan loss and cartilage damage, and increased type II collagen levels in cartilage in animal models of OA [43,116]. Human studies evaluating the efficacy of metformin in OA show promising results, particularly in slowing structural progression and reducing pain. One cohort study from the Osteoarthritis Initiative (OAI) reported that patients receiving metformin experienced a slower rate of medial cartilage volume loss over four years and a trend towards a reduced risk of knee joint replacement over six years [126]. Similarly, a study using Taiwan’s National Health Insurance Research Database demonstrated that combining metformin with a COX-2 inhibitor reduced the rate of joint replacement surgery by 25% over ten years [127]. However, a UK database study found no significant association between metformin use and new OA diagnoses [128]. Additionally, metformin showed immunomodulatory benefits by reducing serum levels of pro-inflammatory mediators, such as IL-1β, IL-8, and TNFα, in patients with symptomatic knee OA. A placebo-controlled, double-blind, randomized clinical trial assessing the effect of metformin in patients with symptomatic knee OA and overweight is currently ongoing [129].

## 6. Discussion

This narrative review highlights the multifaceted role of autophagy in OA and underscores its dual nature as both a protective and detrimental mechanism depending on the stage of the disease. In the early stages of OA, autophagy plays a crucial role in maintaining chondrocyte homeostasis by acting as a cytoprotective mechanism. During this phase, autophagy mitigates the effects of low-grade inflammation and oxidative stress, which are key features of the early inflammatory environment. The activation of autophagy-related proteins helps remove damaged mitochondria and misfolded proteins, thereby preserving chondrocyte viability and delaying cartilage degeneration [111].

A major hallmark of OA that has not been discussed in the previous sections is cellular senescence, in which autophagy was shown to play a critical role. Senescent chondrocytes accumulate in OA cartilage and exhibit the senescence-associated secretory phenotype (SASP), characterized by the secretion of inflammatory cytokines (e.g., IL-6, IL-8), MMP-13, and ROS, exacerbating cartilage degradation [130]. Studies of human OA cartilage have shown that autophagy markers, such as LC3 and beclin1, are significantly reduced in diseased tissue compared to healthy cartilage, contributing to increased senescence and apoptosis [66]. Reduced autophagy disrupts the balance between cartilage degradation and repair mechanisms, leading to accelerated aging-related senescence in chondrocytes [130]. Dysfunctional autophagy exacerbates SASP by allowing the accumulation of cellular debris, further driving inflammation and cartilage matrix breakdown [131]. Chen et al. demonstrated that the methyltransferase METTL3 mediates m6A modification of ATG7, a key autophagy-related gene, the epigenetic regulation being crucial for maintaining autophagy balance in chondrocytes. In OA, reduced METTL3 levels led to suppressed autophagy and increased senescence in chondrocytes, while restoring METTL3 improved cartilage homeostasis by alleviating SASP factors. Additionally, METTL3-mediated autophagy regulation was shown to attenuate inflammation and slow OA progression in ex vivo human cartilage models [132].

**Figure 1:**
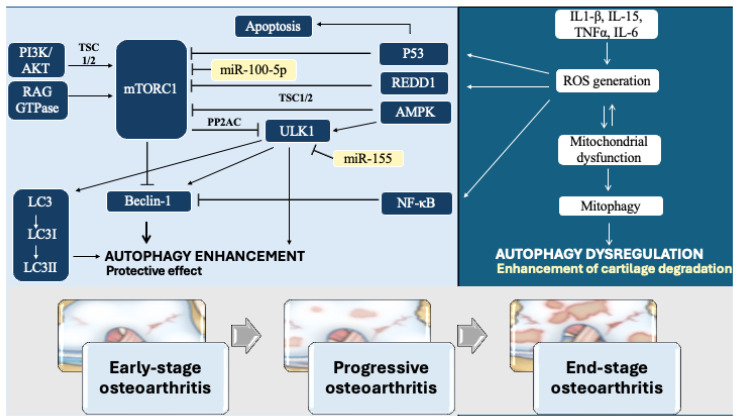
Key signaling pathways associated with autophagy regulation in OA. AMPK, AMP-activated protein kinase; AKT, Protein kinase B; Beclin1, Autophagy-related protein; IL-1β, Interleukin 1 beta; IL-15, Interleukin 15; IL-6, Interleukin 6; LC3, Microtubule-associated proteins 1A/1B light chain 3; LC3I/LC3II, Forms of LC3 during autophagy; miR-100-5p, MicroRNA-100-5p; miR-155, MicroRNA-155; mTORC1, Mechanistic target of rapamycin complex 1; NF-κB, Nuclear factor kappa-light-chain-enhancer of activated B cells; P53, Tumor protein P53; PI3K, Phosphoinositide 3-kinases; PP2AC, Protein phosphatase 2A catalytic subunit; RAG GTPase, Ras-related GTPase; REDD1, regulated in development and DNA damage responses 1; ROS, reactive oxygen species; TNFα, Tumor necrosis factor alpha; TSC1/2, Tuberous sclerosis complex 1/2; ULK1, Unc-51-like autophagy activating kinase 1; →, stimulation; 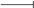, inhibition.

Pharmacological interventions targeting key signaling pathways, such as mTOR [112], AMPK/ULK1 [110], and NF-κB/STAT3 [114], have shown consistent results in enhancing autophagy, reducing inflammation, and preventing cartilage degradation. These pathways are critical in regulating autophagy and highlight the potential for autophagy as a therapeutic target in early-stage OA. The ULK complex, as a central regulator of multiple autophagy pathways, emerges as a particularly promising drug target. Its modulation by upstream signals from mTOR and AMPK (Figure 1) places it at a critical node for controlling autophagy in different cellular contexts, making it a valuable focus for drug development aimed at slowing OA progression [43,105].

However, in advanced stages of OA, excessive or dysfunctional autophagy can become problematic, leading to an accumulation of autophagosomes and impaired autophagic flux [35]. This dysfunction can aggravate chondrocyte death and contribute to cartilage erosion, exacerbating joint destruction. The interplay between autophagy and apoptosis becomes more pronounced in this phase, where autophagy-related stress releases factors such as apoptosis-inducing factors, triggering chondrocyte apoptosis and accelerating joint damage [31,32]. This shift in the role of autophagy—from a survival mechanism to one that promotes cell death—suggests that while enhancing autophagy may be beneficial in early-stage OA, careful modulation is needed in advanced stages to avoid adverse effects.

A significant limitation in developing therapies that modulate autophagy in OA is the potential for off-target effects. As autophagy is a fundamental cellular process involved in maintaining homeostasis in various tissues, its systemic modulation could lead to unintended consequences in other organs, including the liver, muscles, and immune system [7]. For instance, therapies that inhibit mTOR to enhance autophagy in chondrocytes might simultaneously impact metabolic [133] or immune functions [134], potentially leading to harmful side effects. This highlights the importance of developing targeted delivery systems, such as localized intra-articular injections, to specifically modulate autophagy in joint tissues while minimizing systemic exposure. Without this level of precision, the risks associated with systemic side effects may outweigh the therapeutic benefits for OA.

## 7. Conclusions

Autophagy plays a dynamic role in OA, serving as both a protective mechanism and a contributor to cellular dysfunction. The therapeutic modulation of autophagy, particularly through agents like metformin, offers a promising approach to managing OA. However, a deeper understanding of autophagy’s regulation in chondrocytes is essential for developing targeted therapies that can effectively slow OA progression while minimizing the risk of autophagic cell death in later stages of the disease.

## Figures and Tables

**Table 1:** In vivo studies assessing the efficacy of therapeutic interventions.

Animal Strain	Substance (Dose & Administration)	Treatment Duration	Effects of Treatment *	Conclusion	References
Sprague Dawley rats	Bilobalide Oral administration 5 mg/kg/day or 10 mg/kg/day	6 weeks	- alleviated post-traumatic cartilage damage and reduced joint pain - inhibited the production of iNOS, COX-2, and MMP13 - increased LC3 expression - reduced p62 expression - decreased serum levels of cartilage degradation markers CTX-II, COMP, and C2C	Inhibition of mTOR signaling promotes autophagy, reducing cartilage degradation and in OA	[106]
Sprague Dawley rats	Glabridin Intra-articular injection 1 mg/kg, 5 mg/kg, and 10 mg/kg × 2/week	4 or 8 weeks	- reduced OA-induced pain and improved the weight-bearing asymmetry - reduced cartilage erosion and proteoglycan loss - increased LC3-II - decreased p-mTOR levels - decreased MMP13 and ADAMTS5 levels - increased collagen II levels		[107]
Sprague Dawley rats	Four-octyl itaconate Intra-articular injection 100 μM × 1/week	8 weeks	- reduced cartilage degradation - decreased levels of MMP3, MMP13, IL-6, and TNF-α in synovial fluid - enhanced levels of LC3 and beclin1 - reduced levels of Bax and cleaved caspase-3 - reduced phosphorylation levels of PI3K, AKT, and Mtor	Enhancement of autophagy via inhibition of PI3K/AKT/mTOR pathway promotes autophagy, reduces inflammation and prevents cartilage degradation.	[100]
Sprague- Dawley rats	β-ecdysterone Subcutaneous injection 0.6 mg/kg, 0.8 mg/kg, and 1 mg/kg × 2/week	4 weeks	- reduced the levels of IL-1β, IL-6, NO, and TNF-α - increased the expression of LC3II/LC3I - reduced the expression of caspase-3 - decreased levels of p-PI3K, p-AKT, and mTOR		[101]
C57BL/6 mice	Mulberroside A Intra-articular injection 40 mM × 1/week	8 weeks	- alleviated cartilage destruction - increased aggrecan, collagen II, and SOX9 levels - reduced MMP13 and ADAMTS5 expression. - reduced levels of p-PI3K, p-AKT, and mTOR.		[102]
Sprague Dawley rats	Quercetin Intra-articular injection 6 or 8 μM × 1/week	6 weeks	- inhibited cartilage degeneration - decreased MMP13 expression - increased collagen II, aggrecan as well as TSC2 and LC3BII levels - decreased RHEB, p-mTOR, p-ULK1 and p62 levels of expression	Quercetin alleviated knee OA progression via the TSC2/RHBE/mTOR signaling pathway.	[113]
C57BL/6 male mice	Shikonin Intraperitoneal injection 2 mg/kg every two days.	12 weeks	- lowered OARSI scores - increased LC3 II/I ratio, beclin1, p-AMPK expression levels - increased aggrecan and collagen II levels - decreased expression of p62, MMP1 and ADAMTS5	Promoting autophagy and inhibiting apoptosis and inflammatory pathways inhibits OA progression.	[103]
C57BL/6 mice	Calcitriol Dose not specified. Intraperitoneal injection × 1/week.	8 weeks	- reduced cartilage degradation, subchondral bone thickening, and loss of proteoglycans - increased the phosphorylation of AMPK - decreased p-mTOR levels - increased the LC3II/LC3I ratio, beclin1 expression, while reducing that of p62.	Promoting autophagy through the AMPK/mTOR signaling pathway modulates inflammation and reduces cartilage degradation.	[105]
C57BL/6 mice	Metformin Oral administration 100 mg/kg/day and 200 mg/kg/day.	10 weeks	- reduced proteoglycan loss and cartilage damage - reduced MMP13 and MMP3 expression levels - increased p-AMPK levels - reduced p-S6 levels		[43]
C57BL/6 mice	Metformin Intra-articular injection 1.65 g/mL, every three days	8 weeks	- increased type II collagen levels in cartilage - increased p-AMPK and p-SIRT1 protein levels - decreased MMP13 and cleaved caspase-3 expression levels	Activation of the SIRT1/AMPK-mediated autophagy pathway mitigates cartilage degradation.	[116]
C57BL/6mice	Trehalose Oral administration—2% and 5% trehalose solutions, given in the drinking water.	8 weeks	- decreased proteoglycan loss, cartilage erosion, and lowered OARSI scores; - decreased the levels of expression of p62, cleaved caspase-3, -9, -12, cytochrome C, CHOP, SOD2, Bax - increased LC3II/LC3I ratio - increased the levels of expression of BNIP3, PGAM5, Bcl-2 - increased p-AMPK and p-ULK levels	Activation of the AMPK/ULK1 pathway reduced oxidative stress-induced apoptosis and ER stress in chondrocytes and enhanced autophagy independently of mTOR.	[111]
Sprague Dawley rats	Osthole Intraperitoneal injection 1 mg/kg/day	10 days	- increased aggrecan and collagen II levels - upregulated p-AMPK, p-ULK1, and LC3 II/I levels - decreased expression of p62, MMP13 and ADAMTS5	Activation of AMPK/ULK1 signaling pathway	[110]
C57BL/6 male mice	MiRNA 100-5p Intra-articular injection 160 μM × 2/week	4 or 6 weeks	- improved gait abnormalities in OA mice - increased levels of aggrecan and collagen II - reduced MMP3, MMP13, and ADAMTS5 levels of expression - decreased the expression of mTOR and p62 and increased the level of LC3-II	miR-100-5p inhibits mTOR pathway, enhancing autophagy and providing chondroprotection	[112]
Rat strain not specified	MiR-146a-5p antagomir 50 nmol/L intra-articular injection × 1/week	13 weeks	- inhibited cartilage degeneration - increased beclin1, ATG5, LC3, and ATG7 levels of expression - increased the LC3II/LC3I ratio - reduced the expression of caspase-3	Downregulation of miR-146a-5p inhibits apoptosis and enhances chondrocyte autophagy via NUMB.	[95]
C57BL/6J mice	Alantolactone Intra-articular injection 2 mg/kg × 2/week	8 weeks	- increased collagen II levels - decreased protein levels of iNOS, COX2, MMP1, MMP3, MMP13, and ADAMTS5 - reduced levels of p62 and increased conversion of LC3I to LC3II. - reduced p-STAT3 levels and the nuclear translocation of STAT3 - reduced p-IκBα and p-P65 levels	Inhibition of NF-κB/STAT3 pathway enhanced autophagy induced by IL-1β reducing OA progression	[114]
Sprague Dawley rats	Celastrol Intraperitoneal injection 0.5 mg/kg/day or 1 mg/kg/day × 1/day	12 weeks	- increased LC3-II/LC3-I ratio, beclin1 expression - reduced p-IκBα and p-P65 levels - reduced levels of p62, cleaved caspase-3 - reduced TNF-α and IL-6 levels	Enhancement of autophagy and inhibition of apoptosis via NF-κB signaling, reduces inflammation and cartilage degradation	[115]
Sprague Dawley rats	FBXO21-specific knockdown shRNA (30 µL of 1010 PFU/mL) × 3/week	2 weeks	- increased LC3 II/I ratio and beclin1 levels - increased aggrecan and collagen II levels - decreased MMP3, cleaved caspase-3 levels of expression	FBXO21 promotes autophagy and reduces matrix catabolism and cell death reducing OA-related degeneration	[104]

Legend: ADAMTS5, A disintegrin and Metalloproteinase with Thrombospondin Motifs; AMPK, AMP-Activated Protein Kinase; ATG, Autophagy Related; Bax, Bcl-2-Associated X Protein; Bcl-2, B-Cell Lymphoma; CHOP, C/EBP Homologous Protein; COX-2, Cyclooxygenase-2; C2C, Cartilage Oligomeric Matrix Protein Breakdown Products; COMP, Cartilage Oligomeric Matrix Protein; CTX-II, C-telopeptide of Type II Collagen; IL, Interleukin; iNOS, Inducible Nitric Oxide Synthase; LC3, Microtubule-associated proteins 1A/1B light chain 3B; MMP, Matrix Metalloprotease; mTOR, Mammalian Target of Rapamycin; NO, Nitric Oxide; OA, Osteoarthritis; OARSI, Osteoarthritis Research Society International; PGAM5, Phosphoglycerate mutase family member 5; PI3K, Phosphoinositide 3-Kinases; p-, phosphorylated; RHEB, Ras Homolog Enriched in Brain; SD; Sprague Dawley; SOD2, Superoxide Dismutase 2; SOX9, SRY-Box Transcription Factor 9; STAT3, Signal Transducer and Activator of Transcription 3; TNF-α, Tumor Necrosis Factor Alpha; TSC2, Tuberous Sclerosis Complex 2. * Biomarkers were measured in cartilage or synovial fluids.

## Data Availability

All data generated or analyzed during this study are included in this published article.
